# An autologous dendritic cell vaccine polarizes a Th-1 response which is tumoricidal to patient-derived breast cancer cells

**DOI:** 10.1007/s00262-018-2238-5

**Published:** 2018-10-03

**Authors:** Michele Tomasicchio, Lynn Semple, Aliasgar Esmail, Richard Meldau, Philippa Randall, Anil Pooran, Malika Davids, Lydia Cairncross, David Anderson, Jennifer Downs, Francois Malherbe, Nicolas Novitzky, Eugenio Panieri, Suzette Oelofse, Rolanda Londt, Thurandrie Naiker, Keertan Dheda

**Affiliations:** 1 Division of Pulmonology and UCT Lung Institute, Department of Medicine, Centre for Lung Infection and Immunity, Groote Schuur Hospital, University of Cape Town, Old Main Building, H46.41, Groote Schuur Drive, Observatory, Cape Town, 7925 South Africa; 2 Department of General Surgery, University of Cape Town and Groote Schuur Hospital, Cape Town, South Africa; 3 Division of Radiation Oncology, Department of Radiation Medicine, University of Cape Town and Groote Schuur Hospital, Cape Town, South Africa; 4 National Health Laboratory Services (NHLS), Groote Schuur Hospital, Haematology, Cape Town, South Africa; 5 Division of Haematology, University of Cape Town, Cape Town, South Africa; 6 Institute of Infectious Diseases and Molecular Medicine, University of Cape Town, Cape Town, South Africa

**Keywords:** Dendritic cell vaccine, Immunotherapy, Breast cancer, Tumour lysate, Autologous primary breast cancer cells

## Abstract

Breast cancer remains one of the leading causes of cancer-associated death worldwide. Conventional treatment is associated with substantial toxicity and suboptimal efficacy. We, therefore, developed and evaluated the in vitro efficacy of an autologous dendritic cell (DC) vaccine to treat breast cancer. We recruited 12 female patients with stage 1, 2, or 3 breast cancer and matured their DCs with autologous tumour-specific lysate, a toll-like receptor (TLR)-3 and 7/8 agonist, and an interferon-containing cocktail. The efficacy of the vaccine was evaluated by its ability to elicit a cytotoxic T-lymphocyte response to autologous breast cancer cells in vitro. Matured DCs (≥ 60% upregulation of CD80, CD86, CD83, and CCR7) produced high levels of the Th1 effector cytokine, IL12-p70 (1.2 ng/ml; *p* < 0.0001), compared to DCs pulsed with tumour lysate, or matured with an interferon-containing cocktail alone. We further showed that matured DCs enhance antigen-specific CD8 + T-cell responses to HER-2 (4.5%; *p* < 0.005) and MUC-1 (19%; *p* < 0.05) tetramers. The mature DCs could elicit a robust and dose-dependent antigen-specific cytotoxic T-lymphocyte response (65%) which was tumoricidal to autologous breast cancer cells in vitro compared to T-lymphocytes that were primed with autologous lysate loaded-DCs (*p* < 0.005). Lastly, we showed that the mature DCs post-cryopreservation maintained high viability, maintained their mature phenotype, and remained free of endotoxins or mycoplasma. We have developed a DC vaccine that is cytotoxic to autologous breast cancer cells in vitro. The tools and technology generated here will now be applied to a phase I/IIa clinical trial.

**Electronic supplementary material:**

The online version of this article (10.1007/s00262-018-2238-5) contains supplementary material, which is available to authorized users.

## Introduction

Cancer remains one of the leading causes of death worldwide with approximately 14 million new cases in 2012 and 8.8 million related deaths recorded in 2015 [1]. Breast cancer represents 14% of the total global cancer-related deaths in females [2]. Breast cancer staging was defined according to standard guidelines [3]. Stage 1 was defined as a tumour < 20 mm in size that was confined to one breast only. Stage 2 was defined as a tumour < 50 mm in size with or without malignant cell invasion of auxiliary lymph nodes and/or lymph nodes near the breastbone. Stage 3 was defined as a tumour > 5 cm which had spread to auxiliary lymph nodes and/or lymph nodes near the breastbone or any size tumour that has spread to other areas within the breast. Stage 4 was defined as breast cancer that has metastasised beyond the breast to the lungs, lymph nodes, skin, bones, liver or brain.

Surgery for the most part is an effective treatment method, but its success is limited to the early stages of the disease before breast cancer has metastasised. Other forms of therapy, including chemotherapy, are partially effective but are associated with substantial and severe adverse events. Thus, new therapeutic options are urgently required.

DC are potent antigen presenting cells, which prime and activate T-cells during microbial or viral infection [4]. DCs offer an attractive immunotherapeutic option because they can be primed with different antigens in vitro to target different diseases in vivo. Various TLR agonists (e.g. TLR-3 [Ampligen® and Poly I:C] and TLR-7/8 [R848]) have been used to mature DCs in vitro for use as immunotherapeutic agents against malignant melanoma [5], prostate cancer [6], malignant glioma [7] and renal cancer [6]. These DCs have the ability to express bioactive IL-12p70, IFN-α, IFN-γ, and TNF-α [8–10], indicating that they can support anti-tumour Th1 responses. By contrast, earlier DC vaccines could cross-present tumour antigens but lacked either co-stimulatory ability or lymph node homing capacity, or they produced low levels of IL12-p70, which is essential for Th1 polarising immunity [11]. The ability of DCs to produce IL-12p70 has been shown to directly translate to clinical benefits in vivo [12–14].

Over the last 5 years’ clinical trials have been conducted involving different cancers using different DC vaccines, which support the efficacy of DCs as immunotherapeutic agents [15–17]. Notably, these studies evaluated vaccines developed using cancer cell lines. However, in contradistinction to cell lines there is considerable antigenic variability amongst tumours from different individuals with the same type of cancer. For example, the commonly used MCF-7 breast cancer cell line does not expresses some antigens that are highly expressed in 75–80% of breast cancers encountered in clinical practice [18–21]. This may result in poor vaccine efficacy because of tumour antigen heterogeneity [4, 16]. To address this issue, we tested DC vaccine efficacy to the patient’s own tumour cells in vitro (and to our knowledge the first study to do so) by recruiting female patients with stage 1, 2 and 3 breast cancer.

We show that we can optimally mature patient-derived DCs in vitro with tumour-specific lysate, Ampligen®, an IFN-containing cocktail (IFN-α, IFN-γ, IL-1β, CD40L) and R848. We cultured and used patient-derived primary breast cancer cells as “targets” to test the efficacy of the DC vaccine in vitro. The mature DCs had the ability to prime effector cells, which resulted in Th1 cytotoxic CTL-mediated killing of the patient’s own breast cancer cells in vitro. We further show that the mature DCs were sterile, endotoxin/mycoplasma free, and they maintained their mature phenotype and high viability 2 months’ post-cryopreservation.

## Methods

### Study site and population

Women undergoing surgery as the standard of care at Groote Schuur Hospital in Cape Town, South Africa were identified as potential participants. Patients over the age of 18 and diagnosed with stage 1, 2, or 3 breast cancer were recruited to the study and written informed consent was obtained. A clinical research form was completed for every patient recruited, which indicated age, reproductive status and medication status. Exclusion criteria included (i) patients undergoing immunotherapy, (ii) patients receiving immunosuppressive medication (iii) patients on hormonal treatment for breast cancer, (iii) active second malignancies, i.e. any malignancy not treated with curative intent within the last 5 years, (iv) patients with auto-immune disease, (v) any substance abuse. All participants agreed to donate a piece of malignant breast tissue and to undergo a leukapheresis procedure at a later date.

### Autologous breast cancer cell culture

Approximately, ten 10 mm × 2 mm biopsy specimens (mean weight = 244 mg; Table 1) were obtained from the core of each tumour post-surgery (mean size = 22 mm × 21 mm [w × d]; Table 1) and the tissue was cut into 1 mm by 1 mm pieces and separated into two equal portions; for autologous breast cancer cell culture and for the generation of a tumour lysate. The autologous primary cells were isolated from the biopsy sample using Collagenase II according to the manufacturers specifications (Ambion, USA). The cells were washed and seeded in the appropriate culture vessel at 100% confluency in DMEM/F12 medium containing 10% human A/B serum (Western Province Blood Transfusion Services, South Africa), 100 IU penicillin/streptomycin, 0.1 mM sodium pyruvate (Lonza, Switzerland), 10 µg/ml insulin, 10 µg/ml transferrin, 10 µM ethanolamine, 10 ng/ml selenium (DMEM/F12-10; Sigma–Aldrich, Germany) and 100 nM estradiol (Sigma–Aldrich, Germany). After 2 days incubation at 37 °C the medium was replaced without estradiol, but with 100 nM cortisol (Sigma–Aldrich, Germany) to prevent fibroblast growth [22, 23]. The cells were continually cultured until 100% confluency. They were lifted with trypsin/EDTA (Lonza, Switzerland) and cultured in larger culture vessels until the cells were confluent (~ 2 × 10^7^ cells in total) in a T175 tissue culture flask (Greiner, Germany). The cells were cultured in DMEM/F12-10 without cortisol one week prior to co-culture with the effector T-cells. We demonstrated that we had the ability to culture the primary breast cancer cells for several weeks. Each culture was cryopreserved in DMEM/F12 with 40% human A/B serum and 10% DMSO as indicated below.

**Table 1:** Demographic data of the cohorts used in study and phenotypic characterisation of the primary breast cancer cells

Patient ID	Age	Race	Stage	Tumour size (w×d) [mm]	Tumour biopsy weight (mg)	Invasive	Antigens expressed (IHC) Detected (Yes/No)			Antigens expressed (FC)				HLA-type
							ER	PR	HER-2	Ep-CAM	CD49f	MUC-1	HER-2	
PC001	44	African	3	21×20	253	Yes	No	Yes	Yes	+	+++	+++	+++	A30, A68
PC003	58	Mixed	2	25×20	404	No	No	No	IC	++	+	+++	++++	A02, A30
PC004	71	Mixed	3	20×15	186	No	No	Yes	Yes	+++	++++	+++	++++	A30, A33
PC007	58	Mixed	3	20×15	220	No	No	Yes	Yes	++	+++	+++	++++	A03, A11
PC009	39	Mixed	2	20×15	345	No	Yes	Yes	Yes	+	++	+++	++++	A01, A03
PC010	44	Mixed	2	30×30	192	No	Yes	Yes	Yes	+	+	+++	++++	A02, A66
PC011	42	Mixed	1	20×20	116	No	Yes	Yes	Yes	++	+	+++	++++	A02, A24
PC012	48	Mixed	2	35×30	224	No	Yes	Yes	Yes	ND	ND	ND	ND	A02, A11
PC013	41	African	3	40×45	165	Yes	No	No	Yes	+++	++	+++	++++	A02
PC015	38	Mixed	2	11×12	216	Yes	No	No	Yes	+++	++	+++	++++	A02, A03
PC016	44	Mixed	3	7×16	185	Yes	Yes	Yes	Yes	+++	++	+++	++++	A02, A26
PC021	41	Mixed	3	20×16	320	No	Yes	Yes	Yes	+++	+++	++++	++++	A02, A24

*IHC* immunohistochemistry, *FC* flow cytometry, *HLA* human leukocyte antigen, *+* denotes 0–25% expression, *++* denotes 25–50% expression, *+++* denotes 50–75% expression, *++++* denotes 75–100% expression, *IC* inconclusive. *ND* not determined

### Preparation of tumour lysate

For the generation of a tumour lysate, the tumour tissue was homogenised on ice with a tissue ruptor (Qiagen, Germany). The homogenate was subjected to 5 freeze thaw cycles, which involved snap freezing in liquid nitrogen followed by incubation at 37 °C for 5 min. Total protein was determined using a standard Bradford assay (BioRad, USA) as per the manufacturer’s instruction.

### Culture conditions to obtain mature DCs

Each patient underwent a leukapheresis procedure using the Colbe Spectra Optia® Apheresis System (Terumo BCT, USA). Following leukapheresis the monocytes (~ 2 × 10^7^ cells) were purified by plastic adherence and differentiated into immature DCs with CellGenix DC medium (CellGenix, Germany) containing 100 µg/mL IL-4 and GM-CSF (Prospec Bio, Israel) for 5 days at 37 °C. After 5 days, immature DCs were pulsed with or without 100 µg/ml of tumour-specific lysate for 6 h at 37 °C and then matured with or without or with different combinations of 100 µg/mL Ampligen® (Hemispherx Biopharma, USA), an IFN-containing cocktail (25 ng/mL IFN-γ, 10 ng/mL IFN-α, 10 ng/mL IL1-β, 1 µg/mL CD40L; Prospec Bio, Israel) and 2.5 µg/mL R848 (InvivoGen, USA) for 42 h at 37 °C. Supernatants derived from the mature DCs were stored at − 80 °C for IL12-p70 analysis by the ELISA.

### Phenotypic assessment of the mature DCs using flow cytometry

Immature and mature DCs were stained with HLA-DR PerCP/Cy5.5, CD40 FITC, CCR7 PE, CD80 PE/CY7, CD86 PE-Dazzle 594 and CD83 APC (Biolegend, USA). The cells were acquired using a LSRII flow cytometer (Beckton Dickinson, USA) and analysed using FloJo software (version 10.1; Treestar, USA). Dead cells were gated out of the scatter plots prior to analysis and negative gates were set using mean fluorescence one (MFO) controls.

### Confocal microscopy

Monocytes, immature DCs and mature DCs were prepared as indicated previously. The cells were allowed to adhere to 3-aminopropyltriethoxysilane (APES; Sigma, Germany) coated slides overnight at 37 °C. The next day the cells were stained with or without or in combination with CD14 PE/Cy7, CD40 FITC and or CD83 APC (Becton Dickinson, USA) and the slides were mounted in Mowiol (Calbiochem, USA) containing n-propyl gallate (Sigma–Aldrich, Germany) as anti-fading agent. Confocal microscopy was performed with a Zeiss Axiovert 200M LSM 510 Meta NLO Confocal Microscope using the 40X water immersion objective and the 63X oil-immersion objective.

### Cytospin, haematoxylin, eosin staining and light microscopy

Monocytes, immature DCs and mature DCs were concentrated onto glass slides using cytospin (Cytospin 3, Shandon, UK) and stained with haematoxylin and eosin (Merck, Germany) using a standard technique. The slides were viewed using a Nikon light microscope with the 100x oil-immersion objective.

### Immunohistochemistry of the breast cancer biopsies

Immunohistochemistry of the biopsy samples using antibodies directed to the estrogen receptor (ER), progesterone receptor (PR) and human epidermal growth factor receptor (HER-2) were performed by the National Health Laboratory Services (NHLS) at Groote Schuur Hospital, Cape Town, South Africa.

### Phenotypic characterisation of the autologous breast cancer cells using flow cytometry

The autologous breast cancer cells were stained with HER-2 PE, epithelial cell adhesion molecule (Ep-CAM) PE-Dazzle 594, mucin-1 (MUC-1) PE-Cy7 and integrin alpha 6 (CD49f) APC (Biolegend, USA) as recommended by the manufacturer. The cells were acquired on the LSRII flow cytometer and the data were analysed as indicated previously.

### IL12-p70 ELISA

The expression of IL12-p70 was determined using a standard ELISA technique from the culture supernatants obtained above according to the manufacturer’s specifications (Mabtech, Sweden).

### Generation of effector cells

Mature DCs prepared as previously described, were co-cultured with PBMCs as described by Koido et al. [24]. Briefly, mature DCs were co-cultured with PBMCs at a ratio of 1:10 in RPMI (Lonza, Switzerland) medium supplemented with 10% human A/B serum (Western Province Blood Transfusion Services, South Africa), 2 mM L-glutamine, 25 mM HEPES, 0.1 mg/mL sodium pyruvate, 100 IU/ml penicillin and 100 mg/ml streptomycin (R-10; Sigma, Germany). After 3 days of culture the medium was replaced with fresh medium containing 10 U/ml IL-2 (Roche, Switzerland). The cells were then cultured for an additional 4 days at 37 °C to generate the effector cells.

### Determination of cytotoxicity and CTL–induced cell death of autologous breast cancer cells

The autologous breast cancer cells were washed then detached with Accumax (Innovative Cell technologies, USA) as indicated by the manufacturer. The autologous breast cancer cells were then co-cultured with the effector cells (generated as indicated) at various ratios of 2:1, 5:1 and 10:1 (effector cells : autologous breast cancer cells). Autologous cells alone served as a negative control. After 4 h of incubation at 37 °C, cytotoxicity was determined using the LDH assay (Cytotoxicity Detection Kit^Plus^ LDH; Roche, Germany) and cell death was measured using 7-aminoactinomycin D (7-AAD; Becton Dickinson, USA) by flow cytometry.

### Tetramer assay

The MHC-1-specific tetramers used in the current study were HLA-02 positive, therefore, only matched patient samples were analysed for the recognition of HER-2 and MUC-1 antigens by the TCRs of CD8 + T-cells. Effector cells were stained with MUC-1 PE tetramer, HER-2 APC tetramer (MBL, USA), CD8 FITC (Becton Dickinson, USA) and Zombie NIR (Biolegend, USA) as recommended by the manufacturer then acquired by flow cytometry and analysed as previously indicated.

### Cryopreservation, sterility and endotoxin/mycoplasma determination

Mature DCs were cryopreserved in R-10 containing 10% DMSO (Sigma, Germany) and 40% human A/B serum at a concentration of 1 × 10^7^/ml at − 80 °C. After 2 months’ cryopreservation, the viability was assessed using trypan blue staining and the maturation phenotype by flow cytometry.

Routine bacterial and mycological sterility testing was conducted on every batch of mature DCs by the NHLS at Groote Schuur Hospital, Cape Town, South Africa. The levels of endotoxin and mycoplasma was determined using the Endpoint Chromogenic Limulus Amoebocyte Lysate (LAL) Assay (ThermoFisher, Scientific, USA) or the MycoAlert™ detection kit (Lonza, Germany) according to the manufacturer’s specifications, respectively.

### Statistics

Data were analysed for statistical significance by one-way Anova with Dunnets post-test or a Wilcoxon signed rank paired *t* test using GraphPad Prism software (version 6.0; GraphPad Software, USA), where *, **, ***, **** indicate *p* < 0.05, *p* < 0.01, *p* < 0.005, *p* < 0.0001, respectively.

## Results

### Patients and samples

**Fig. 1:**
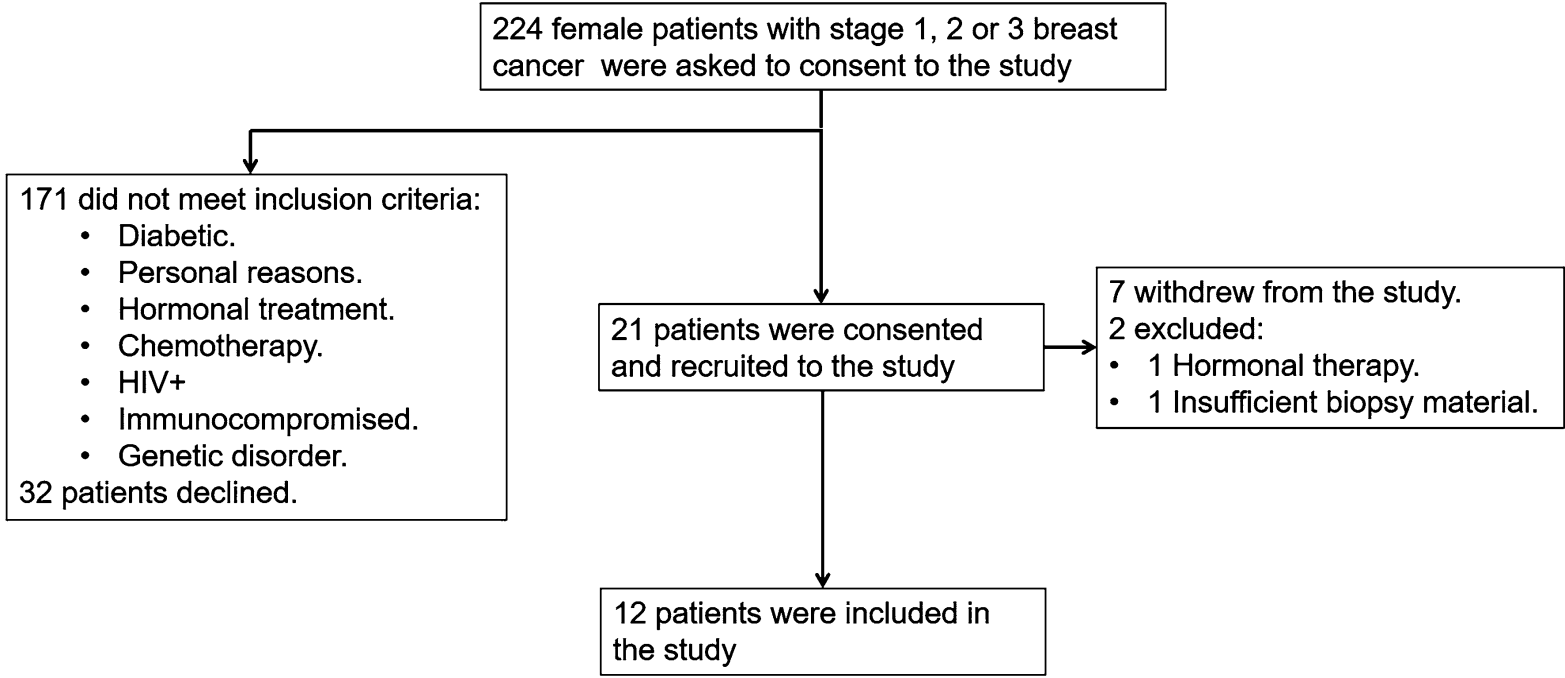
Patient recruitment plan for the DC vaccine breast cancer preclinical trial. Two hundred and twenty-four patients were asked to consent to the study. Two hundred and three patients were not included in the study because they either declined or they did not meet the inclusion criteria. Of the remaining 21 patients who consented to the study a further 9 were excluded or withdrew from the trial. In total 12 patients were included in the current preclinical trial

Two hundred and twenty-four female patients with stage 1, 2 and 3 breast cancer were asked to consent to the study (Fig. 1). Thirty-two patients declined and 171 did not meet the inclusion criteria. Of the remaining 21 a further 7 withdrew and 2 were excluded; one failed to disclose her hormonal treatment and the other did not have enough biopsy material to complete the assays. The remaining 12 female patients were included in the preclinical study.

The demographics of the study cohorts are shown in Table 1. The median age of the patients was 47 years. The mean size of the tumours and weight of the biopsy specimens were 22 mm × 21 mm and 244 mg, respectively (Table 1). The patients were more likely to have non-invasive stage 3 breast cancer and the tumours expressed different breast cancer antigens including, the ER and PR as determined by immunohistochemistry (IHC; Table 1). All the tumours were HER-2 positive. The autologous breast cancer cells all expressed high levels of MUC-1/HER-2 and variable levels of the epithelial (Ep-CAM) and epithelial progenitor (CD49f) markers as determined by flow cytometry (Table 1). Each patient was HLA typed to match them to the HER-2 and MUC-1 tetramers (HLA-A02) used in the study. All the patients had normal blood counts prior to leukapheresis (data not shown).

### DCs from breast cancer patients pulsed with tumour-specific lysate and matured with Ampligen®, an IFN-containing cocktail and R848 or IFN-containing cocktail alone express high levels of key co-stimulatory molecules

**Fig. 2:**
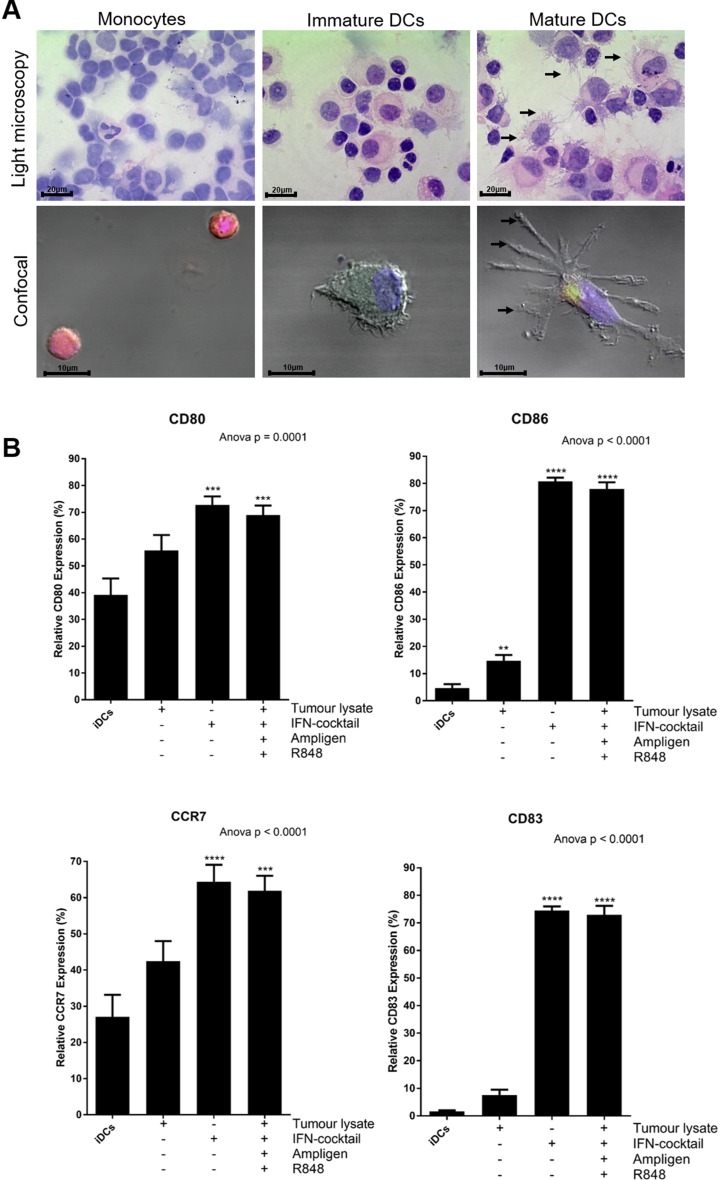
DCs from patients with breast cancer, pulsed with tumour-specific lysate and matured with Ampligen®, an IFN-containing cocktail and R848 and or with IFN-containing cocktail only express higher levels of co-stimulatory molecules compared to immature DCs or DCs pulsed with tumour-specific lysate only. Immature DCs were differentiated from monocytes, incubated in CellGenix DC complete medium with or without 100 µg/mL tumour-specific lysate for 6 h at 37 °C. The cells were then matured with or without or in combination with 100 μg/ml Ampligen®, an IFN-containing cocktail (10 ng/mL IFN-α, 25 ng/mL IFN-γ, 1 µg/mL CD40L and 10 ng/mL IL-1β), and/or 2.5 µg/mL R848 for 42 h at 37 °C. The monocytes, immature DCs and mature DCs were subjected to a haematoxylin and eosin stain (**a**) or were stained with CD14 PE-CY7 (monocytes), CD40 FITC (immature and mature DCs) and or CD83 APC (mature DCs; **A**) for confocal microscopy. The maturation phenotype was also determined by flow cytometry (**b**). Arrows (**→**) show dendrites being expressed on the surface of mature DCs. Data were analysed for statistical significance by one-way Anova with Dunnett’s post-test, where **, *** and **** indicate *p* < 0.01, *p* < 0.005 and *p* < 0.0001, respectively. Each of the treatments were compared to the control group (iDCs). Error bars represent standard deviation. Light microscopy magnification: ×100 (oil immersion); scale bars = 20 µm. Confocal magnification: ×63 (oil immersion); scale bars = 10 µm. iDCs = immature DCs

In optimisation experiments we showed that Ampligen®, an IFN-containing cocktail (IFN-α, IFN-γ, CD40L and IL-1β) and R848, resulted in optimal maturation of the DCs as assessed by the upregulation of HLA-DR, CD40, CD80, CD86, CCR7 and CD83 (data not shown). We also further showed that these mature DCs produced high levels of the Th1 effector cytokine, IL12-p70 (6 ng/1×10^6^/ml; data not shown). The monocytes, immature DCs and mature DCs were morphologically distinct from one another (Fig. 2a). The immature and mature DCs were larger than the monocytes and dendrites were clearly visible on the surface of the cells.

Approximately, a mean of 1 × 10^9^ PBMCs were obtained by leukapheresis for each patient. The PBMCs were washed and monocytes were isolated by plastic adherence. After differentiation into immature DCs using IL-4 and GM-CSF, the cells were pulsed with or without 100 µg/mL tumour-specific lysate for 6 h at 37 °C. The cells were then matured with or without, an IFN-containing cocktail (10 ng/mL IFN-α, 25 ng/mL IFN-γ, 1 µg/mL CD40L and 10 ng/mL IL-1ß), 100 µg/mL Ampligen® and/or 2.5 µg/mL R848 for 42 h at 37 °C. The maturation phenotype was determined by flow cytometry (Fig. 2b). From this point forward IFN-containing cocktail, Ampligen® and R848 will be referred to as full cocktail.

The DCs that were matured with an IFN-containing cocktail only or pulsed with tumour-specific lysate and matured with full cocktail, expressed significantly higher levels of CD40 (*p* < 0.001 or *p* < 0.005, respectively) and HLA-DR (*p* < 0.005) compared to immature DC (data not shown). More importantly, the DCs pulsed with tumour-specific lysate then matured with full cocktail or IFN-containing cocktail alone, expressed significantly higher levels of the key maturation markers, CD80 (69% or 73%, respectively; *p* < 0.005), CD86 (78% or 81%, respectively; *p* < 0.0001), CCR7 (62% or 64%, respectively; *p* < 0.0001) and CD83 (73% or 75%, respectively; *p* < 0.0001), compared to the immature DCs (39% vs 5% vs 27% vs 1.7%, respectively) or DCs pulsed with tumour-specific lysate alone (56% vs 15% vs 42% vs 8%, respectively; Fig. 2B).

### Mature DCs from breast cancer patients produce high levels of the Th1 effector cytokine, IL-12p70

**Fig. 3:**
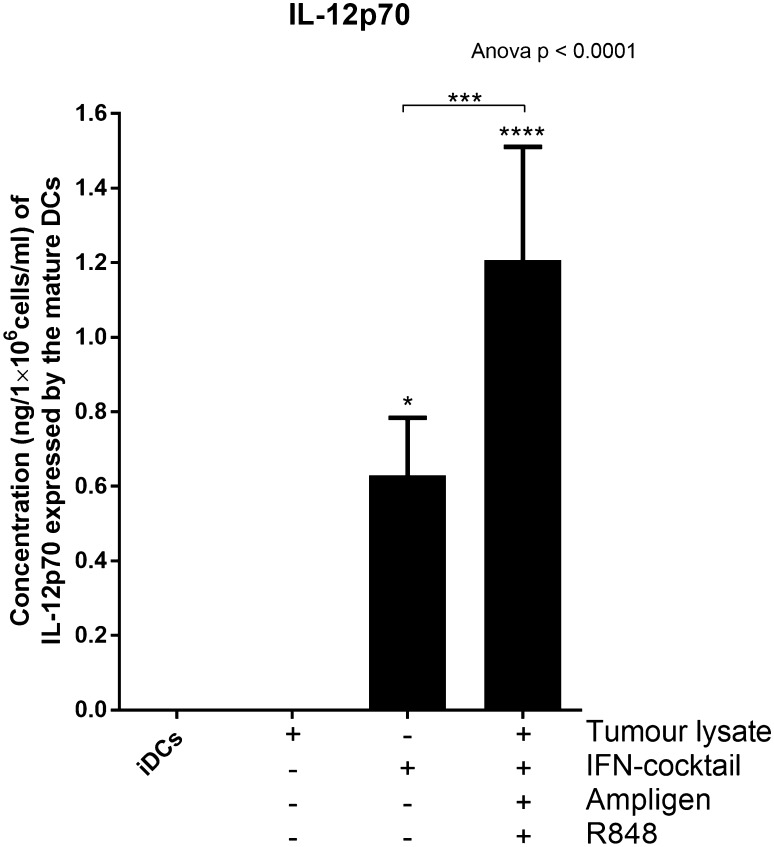
DCs pulsed with tumour-specific lysate and matured with Ampligen®, an IFN-containing cocktail and R848 express higher levels of the Th1 effector cytokines, IL-12p70, compared to the immature DCs (iDCs) or DCs pulsed with tumour-specific lysate only. The level of IL-12p70 from the culture supernatants of the immature DCs or matured DCs was determined using the ELIZAPRO IL-12p70 detection kit from Mabtech as indicated by the manufacturer. Data were analysed for statistical significance by one-way Anova with Dunnett’s post-test or Wilcoxon signed rank paired *t* test, *, ***, **** indicate *p* < 0.05, *p* < 0.005 and *p* < 0.0001, respectively. For Anova each of the treatments were compared to the control group (iDCs). Error bars represent standard deviation

The ability of mature DCs to produce biologically active IL-12p70 is a direct indicator of how clinically effective a DC vaccine can be because it has the ability to activate effector T cells in vivo, that have the potential to drive an anti-tumour response [25–27]. For this reason, we determined the relative expression levels of IL12-p70 from the culture supernatants of the mature DCs using an IL-12p70 ELISA (Fig. 3).

The immature DCs or DCs pulsed with tumour-specific lysate only produced no detectable levels of IL-12p70 (Fig. 3). In contrast, DCs pulsed with tumour-specific lysate and matured with full cocktail expressed high levels of IL-12p70 (1.21 ng/1×10^6^/ml, SD = 0.3–3.7; Fig. 3; p < 0.0001). When the cells were matured with IFN-containing cocktail only the levels of IL-12p70 (0.6 ng/1×10^6^/ml) were significantly different (two-fold less) to the cells that were pulsed with tumour-specific lysate and matured with full cocktail (p < 0.005). This highlights the significant role of Ampligen® and R848 as maturation agents which favour a Th-1 response.

**Fig. 4:**
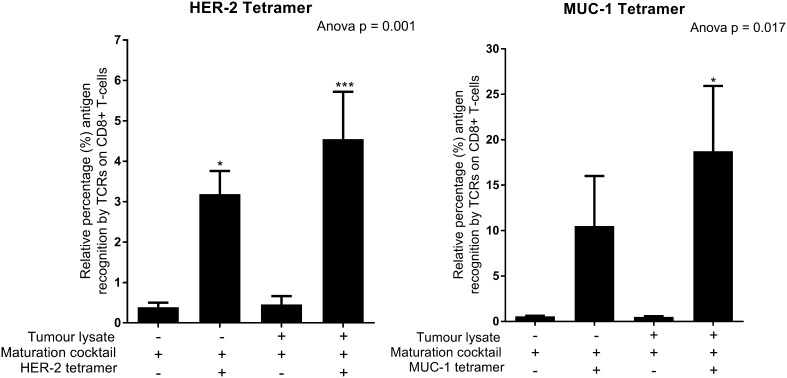
The TCRs of CD8 + T-cells primed with tumour-specific lysate/Ampligen®/IFN-cocktail and R848-matured DCs can recognise HER-2 and MUC-1-specific tetramers. Immature DCs were differentiated from monocytes as indicated previously. DCs were matured and effector cells were generated as indicated in the methods. The cells were stained with CD8 FITC, CD3 PerCP/Cy5.5, HER-2 APC, MUC-1 PE and Zombie NIR according to the manufacturer’s instructions (MBL, USA). The levels of HER-2 and MUC-1 recognised by the TCR on CD8 + T-cells were determined by flow cytometry. Data were analysed for statistical significance by one-way Anova with Dunnett’s post-test where *, *** indicate *p* < 0.05 and *p* < 0.005, respectively. Each of the treatments were compared to the control group (T-cells primed with IFN-cocktail matured DCs not stained with HER-2/MUC-1 tetramer). Error bars represent standard deviation

### The TCRs of CD8 + T-cells primed with tumour-specific lysate and full cocktail-matured DCs can detect HER-2 and MUC-1 antigens on MHC-1 specific tetramers

The MHC-1-specific tetramers were HLA-02 positive hence, it was only possible to analyse patients with the HLA-02 phenotype. The effector cells were stained with the MUC-1 and HER-2 tetramers as indicated in the methods. Both HER-2 (4.5%; *p* < 0.005) and MUC-1 (19%; *p* < 0.05) tetramers detected the TCRs on CD8 + T-cells that were primed with the tumour-specific lysate and full cocktail**-**matured DCs (Fig. 4). A 1.3- and 1.9-fold decrease in HER-2 (3%; *p* < 0.05) and MUC-1 (11%) antigen recognition was observed by the TCRs of the CD8 + T cells primed with DCs matured in the absence of tumour-specific lysate, respectively.

### Cytotoxic-T-cell mediated killing of autologous breast cancer cells with tumour-specific lysate and full cocktail-matured DC primed effector cells

**Fig. 5:**
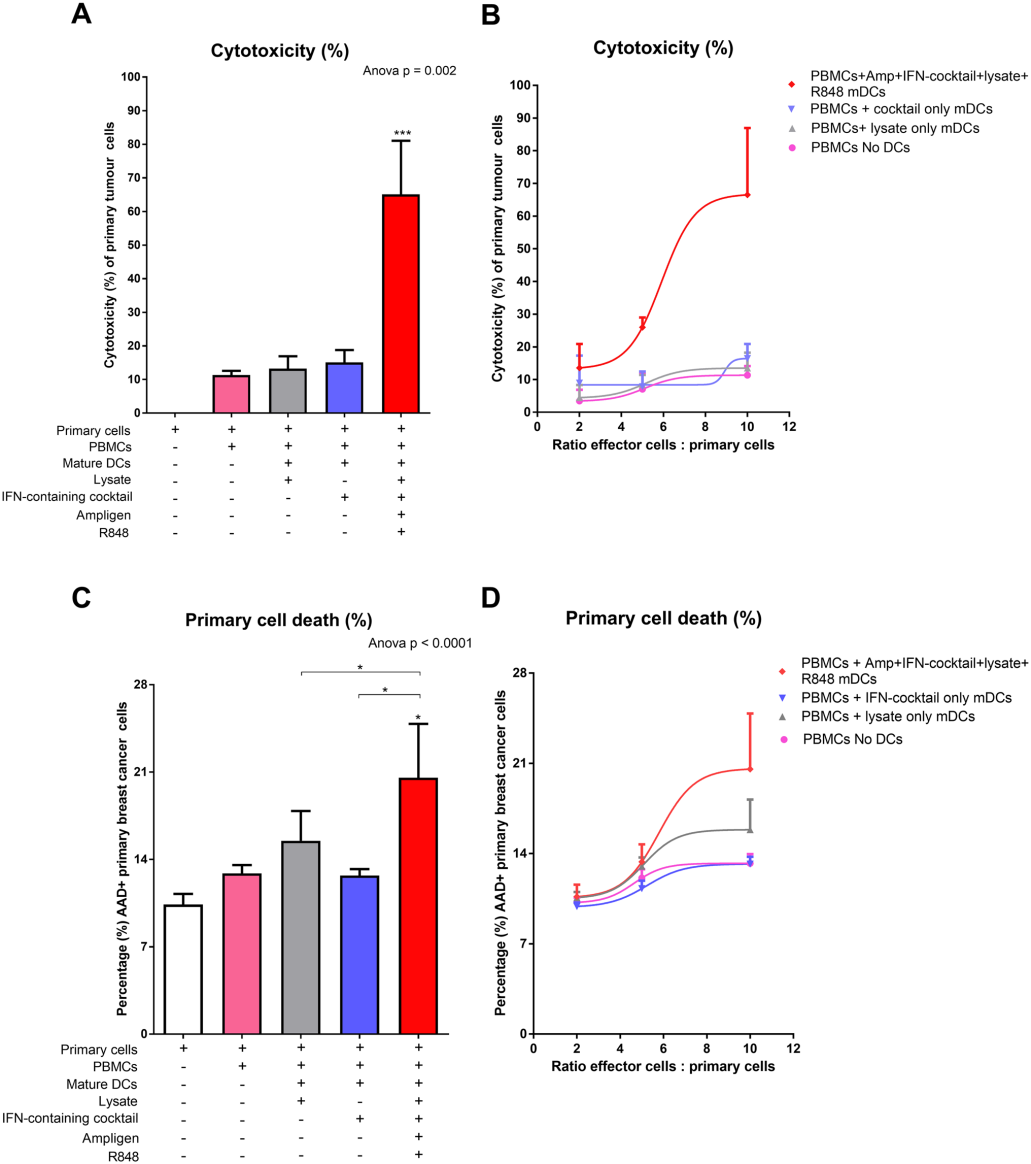
PBMCs from breast cancer patients co-cultured with tumour-specific lysate pulsed and Ampligen®/IFN-containing cocktail/R848-matured DCs results in cytotoxic T-lymphocyte-mediated killing of primary breast cancer cells in vitro. Matured DCs were prepared as indicated in the methods. The matured DCs were then co-cultured with PBMC at a ratio of 1:10 for 7 days at 37 °C. The primary breast cancer cells were incubated with or without the primed PBMCs (effector cells) at a ratio of 1:10 (**a** and **c**) or the primary cells were incubated with the effector T-cells at various ratios indicated (**b** and **d**) for 4 h at 37 °C. Cytotoxicity (**a** and **b**) was determined using the LDH assay (Cytotoxicity Detection Kit^Plus^ LDH; Roche, Germany) and cell death (**c** and **d**) of the primary breast cancer cells was measured by flow cytometry. Data were analysed for statistical significance by one-way Anova with Dunnett’s post-test where *, ***indicate *p* < 0.05 and *p* < 0.005, respectively. Each of the treatments were compared to the control group (primary breast cancer cells incubated in the absence of PBMCs). Error bars represent standard deviation

Next, we wanted to determine if the mature DC-primed effector cells could elicit a CTL response, which was tumoricidal to autologous breast cancer cells in vitro. Effector cells generated as previously described were co-cultured with the autologous breast cancer cells for 4 h. Cytotoxicity of the autologous breast cancer cells was determined using an LDH assay (Fig. 5a, b). In addition, cell death of the autologous breast cancer cells was measured by flow cytometry using 7-AAD (Fig. 5c, d).

When the effector cells were primed with tumour-specific lysate and full cocktail-matured DCs, the median levels of autologous breast cancer cell cytotoxicity were 65% (Fig. 5a). In contrast levels of cytotoxicity were 11%, 13% and 15%, when the effector remained un-primed or were primed with tumour-specific lysate or IFN-containing cocktail only-matured DCs, respectively. We also showed that the levels of cytotoxicity observed were dose-dependent when the effector cells were primed with tumour-specific lysate and full cocktail-matured DCs (Fig. 5b). Once again, the cytotoxic response confirms the importance of Ampligen® and R848 as maturation agents.

Having shown that the effector cells which were primed with tumour-specific lysate, and full cocktail-matured DCs could elicit a cytotoxic response to the autologous breast cancer cells in vitro, we wanted to determine if these cells were tumoricidal in vitro. A two-fold increase (*p* < 0.05) in cytotoxic-mediated autologous breast cancer cell kill was observed with effector cells that were primed with tumour-specific lysate and full cocktail-matured DCs compared to autologous cells not cultured with effector cells (Fig. 5c). We also observed a dose-dependent increase in autologous breast cancer cell kill when the effector cells were primed with tumour-specific lysate and full cocktail-matured DCs (Fig. 5d).

### The tumour-specific lysate and full cocktail-matured DCs were sterile, endotoxin/mycoplasma free and cryopreservation does not affect their maturation phenotype or viability

For the proposed phase I/IIa clinical trial, the vaccine will be administered over a 2-month period. For this reason, we wanted to determine if 2 months of cryopreservation affects the maturation phenotype or viability of the DCs. As shown in Table S1, cryopreservation had no effect on the maturation phenotype of the DCs or on the viability of these cells. The expression levels of the co-stimulatory markers, CD80, CD86, CCR7 and CD83 remained at 84%, 86%, 68% and 77%, respectively. The mean viability was 74% and we show that all the vaccine preparations were sterile and endotoxin/mycoplasma free.

## Discussion

We have developed a Th1-polarising DC vaccine that has high efficacy against patient-derived breast cancer cells in vitro. We show that we can optimally mature DCs in vitro with autologous tumour-specific lysate and a cocktail containing cytokines and TLR agonists. The mature DCs produced high levels of the Th1 effector cytokine IL12-p70. In addition, the TCRs of the mature DC-primed CD8 + T-cells could recognise HER-2 and MUC-1 antigens using a tetramer assay. We further show that these mature DCs could prime effector cells, which resulted in cytotoxic killing of patient-specific autologous breast cancer cells in vitro. To our knowledge this is the first DC vaccine preclinical cancer study that has tested the efficacy of the vaccine against the patient’s own tumour cells in vitro. This is critical to measure vaccine efficacy as breast cancer antigen heterogeneity is high relative to that in cancer cell lines [18, 19, 21].

A major finding was that the IL-12p70-producing mature DCs were proficient in co-stimulating CD8 + antigen-specific tumoricidal responses. This was only observed when Ampligen® and R848 were included during maturation together with tumour-specific lysate and the IFN-containing cocktail. Although the use of DCs as an adoptive cell-mediated therapy for cancer has been widely used [28], our study differs considerably from others as we used autologous breast cancer cells as “target” cells in vitro (and to our knowledge the first to do so). The levels of toxicity reported here are comparable to other studies where the investigators utilised cell lines to test vaccine efficacy [10, 12, 29]. However, the precise levels of cell line-specific cytotoxicity are difficult to measure because of tissue mismatch and induction of an allogenic immune response occurring in tandem, thus underestimating the incremental efficacy of our vaccine. For example the commonly used MCF-7 cell line express very low to undetectable levels of HER-2 [21]. In contrast HER-2 is expressed in some breast cancers that present at the clinic [18, 19]. Therefore, vaccines directed to cell lines may not truly represent the antigenic phenotype of autologous tumours. In this study, all the tumour cells expressed high levels of HER-2, which further highlights the limitations of using cell lines as a model system to test vaccine efficacy.

We showed that the DCs which were pulsed with tumour-specific lysate and matured with full cocktail expressed high levels of CCR7. The high expression levels of co-stimulatory molecules together with CCR7 expression indicate that the DCs not only have optimal T- and natural killer (NK) cell co-stimulatory capacity [30] but also optimal lymph node homing ability [31]. The infiltration of DCs into primary tumour lesions has been associated with significantly prolonged patient survival [32]. A meta-analysis of clinical trials involving DC-based immunotherapy favoured administration of vaccines closest to lymph nodes [6] as only 4–5% of the DCs reach the draining lymph nodes [33]. CCR7 is the dominant receptor involved in the migration of DCs to the draining lymph node, and thus the upregulation of the homing cytokine, CCR7, in our study further supports the use of our DC vaccine as a candidate for therapy.

The individual components included to induce maturation in the current study were chosen to favour type-1 polarisation. Both IFN-γ and CD40L drive high levels of IL-12p70 expression [34] and IL-12p70 and IFN-γ are important for CD8 + T-cell memory development [27]. The TLR agonists, Ampligen® and R848 have been shown to enhance the expression of IFN-γ and IL-12p70 from DCs [9, 35]. Interestingly, R848 induces myeloid-derived suppressor cell (MDSC) differentiation into macrophages and DCs [36]. It is thus an attractive candidate for enhancing the effects of cancer immunotherapy as cells differentiated from MDSCs by the action of R848 exert higher proliferation-inducing activity on antigen-primed T cells compared to untreated MDSCs [36].

We initially pulsed the immature DCs with a tumour lysate prepared from biopsies of breast cancer patients. A meta-analysis from 3444 cancer patients has shown that patients treated with tumour lysate-matured DCs have a more favourable outcome than patients treated with peptide-matured DCs [37]. Electroporation of patient-specific tumour mRNA has been reported to be a more efficient method to enhance MHC class I-mediated antitumor immunity, which mediates a cytotoxic T-cell response without functional deterioration of the DCs [38]. However, in our extensive optimisation studies we found that electroporation of the DCs resulted in suboptimal viability and decreased co-stimulatory molecule expression on the mature DCs (data not shown).

We show that the tumour-specific lysate and full cocktail-matured DCs produced high levels (1.2 ng/1×10^6^/ml/ml) of IL12-p70. A number of human in vitro DC vaccine preclinical trials indicate that IL-12p70 expression is an important predictor of how effective a vaccine can be in an in vivo clinical setting [12, 13] and IL-12p70 has been shown to be indispensable in regulating T-cell effector function [39–42] and NK-induced antitumor responses [43]. In addition mature DCs that produce high levels of IL-12p70 have increased antigen presentation capacity [39] as well as an increased capacity to induce CTL responses to tumour cells [44].

There are limitations to the current study. It was only conducted at one site, so the efficacy of the vaccine was not tested in different clinical settings. However, this was an in vitro preclinical trial and not a phase II or III clinical study. The flow cytometry cell death data may not represent a true reflection of the actual levels of cell lysis and/or death over the 4-h incubation period. The CTL assay is more representative of actual cytotoxicity levels because the assay measures cell membrane lysis over the entire incubation period, while flow cytometry would only measure whole intact dead cells. As a result, the flow cytometric assay would not measure cells that have already lysed or are in the process of lysing due to cytotoxicity. Finally, we were unable to recruit patients with stage 4 breast cancer. However, the immunomodulatory capacity of stage 3 and 4 breast cancer patients would be expected to be similar.

In conclusion, we have developed a DC vaccine to breast cancer, which had potent Th1 polarising ability that is tumoricidal to autologous breast cancer cells in vitro. This has not been reported before and the techniques and methodology used in this preclinical trial will be applied in a phase I safety study.

## Electronic supplementary material

Below is the link to the electronic supplementary material.

Supplementary material 1 (PDF 87 KB)

## Abbreviations

7-AAD: 7-aminoactinomycin D
CD49f: Integrin alpha 6
CD40L: CD40 ligand
Ep-CAM: Epithelial cell adhesion molecule
ER: Estrogen receptor
MUC-1: Mucin-1
NK: Natural killer
Poly I:C: polyinosinic:polycytidylic acid
PR: Progesterone receptor
Th cell: T helper cell
Treg: Regulatory T cell
