# Supplementary material for: An autologous dendritic cell vaccine polarizes a Th-1 response which is tumoricidal to patient-derived breast cancer cells
Source: Cancer Immunol Immunother. 2018 Oct 3;68(1):71–83. doi: 10.1007/s00262-018-2238-5 (PMC6326986; doi:10.1007/s00262-018-2238-5)
Supplement: Supplementary file 1 — Supplementary material 1 (PDF 87 KB) [file 262_2018_2238_MOESM1_ESM.pdf]

Results.

Table S1: Expression of co-stimulatory molecules and viability of mature DCs post 2 months of cryopreservation.

Mean co-stimulatory molecule expression post cryopreservation (%)				GMP testing			
**CD80**	**CD86**	**CCR7**	**CD83**	**Mean viability (%)**	**Sterility (bacterial/mycology)**	**Endotoxins**	**Mycoplasma**
84 (70-90)	86 (72-91)	68 (53-70)	77 (63-85)	74 (60-84)	Yes	No	No

GMP = good manufacturing practice.
